# Predicting the Stone-Free Status of Percutaneous Nephrolithotomy With the Machine Learning System: Comparative Analysis With Guy’s Stone Score and the S.T.O.N.E Score System

**DOI:** 10.3389/fmolb.2022.880291

**Published:** 2022-05-04

**Authors:** Hong Zhao, Wanling Li, Junsheng Li, Li Li, Hang Wang, Jianming Guo

**Affiliations:** ^1^ Shanghai Xuhui Central Hospital, Shanghai, China; ^2^ Zhongshan Hospital, Fudan University, Shanghai, China

**Keywords:** machine learning, prediction, percutaneous nephrolithotomy, stone-free status, Guy’s stone score, S.T.O.N.E score system

## Abstract

**Purpose:** The aim of the study was to use machine learning methods (MLMs) to predict the stone-free status after percutaneous nephrolithotomy (PCNL). We compared the performance of this system with Guy’s stone score and the S.T.O.N.E score system.

**Materials and Methods:** Data from 222 patients (90 females, 41%) who underwent PCNL at our center were used. Twenty-six parameters, including individual variables, renal and stone factors, and surgical factors were used as input data for MLMs. We evaluated the efficacy of four different techniques: Lasso-logistic (LL), random forest (RF), support vector machine (SVM), and Naive Bayes. The model performance was evaluated using the area under the curve (AUC) and compared with that of Guy’s stone score and the S.T.O.N.E score system.

**Results:** The overall stone-free rate was 50% (111/222). To predict the stone-free status, all receiver operating characteristic curves of the four MLMs were above the curve for Guy’s stone score. The AUCs of LL, RF, SVM, and Naive Bayes were 0.879, 0.803, 0.818, and 0.803, respectively. These values were higher than the AUC of Guy’s score system, 0.800. The accuracies of the MLMs (0.803% to 0.818%) were also superior to the S.T.O.N.E score system (0.788%). Among the MLMs, Lasso-logistic showed the most favorable AUC.

**Conclusion:** Machine learning methods can predict the stone-free rate with AUCs not inferior to those of Guy’s stone score and the S.T.O.N.E score system.

## Introduction

Since the first description of the technique in 1976 (Fernstro¨m and Johansson, 1976), percutaneous nephrolithotomy has been widespread for the treatment of renal calculi. It is the golden standard for the treatment of 2-cm kidney stones (Miernik et al., 2014). PCNL’s success rate is between 56% and 96% in various series (Matlaga et al., 2005; Akman et al., 2011; Rosette et al., 2011; Labadie et al., 2015). Many factors contribute to the success of stone clearance including the stone size, location, number, and grade of hydronephrosis, as well as surgeon’s experience. To predict the outcomes after PCNL, several scoring systems have been devised including Guy’s stone score, S.T.O.N.E nephrolithometry system, CROES nephrolithometry nomogram, and S-ReSC score (Al Adl et al., 2020). Guy’s stone score is easy to apply and has been validated in multiple studies. The S.T.O.N.E. score is based on factors determined through CT imaging, which is the currently preferred imaging modality for patients with nephrolithiasis (Noureldin et al., 2015a). The CROES nomogram was developed from data in a large multicenter database and has high statistical power. Determination of the S-ReSC score relies on stone location only, providing a simple approach to grading disease complexity (Noureldin et al., 2015b). Each system has advantages and disadvantages, but several studies suggest that their ability to predict the stone-free rate is comparable (Wu and Okeke, 2017).

Machine learning techniques have been used extensively in the field of clinical medicine, especially when used for the construction of prediction models. The outperformance of ML over conventional data analysis models has been shown in the urology-oncology literature (Hung et al., 2018; Andras et al., 2020; Rodrigo. et al., 2020; Ström et al., 2020).

In predicting post-lithotripsy outcomes with machine learning, there are only three studies published until now (De Perrot et al., 2019; Tayyebe. et al., 2019; Aminsharifi et al., 2020). Aminsharifi et al. (2020) first used the machine learning method for predicting post-PCNL outcomes compared to current scoring systems. They found machine learning-based software was superior in predicting SFS after PCNL, with an AUC of 0.915 compared to 0.615 (GSS) and 0.621 (CROES nomograms) (*p* < 0.01). More than 20 variables of 146 patients were inputted for the training of machine learning in their study. Alireza used a support vector machine (SVM) as the machine learning technique. We know that the machine learning algorithm includes some other methods, such as decision trees, random forests, artificial neural networks, Bayesian learning, Deep Learning, and so on. In this study, we used four machine learning methods (Lasso logistic, random forests, SVM, and Naive Bayes) to predict the SFS of PCNL with the information of 222 patients. We compared the outperformance of ML to Guy’s score and the S.T.O.N.E score system at the same time.

### Patients and Methods

The study was approved by the independent ethics committee of Xu-hui Central Hospital. Between July 2017 and January 2020, 222 patients who underwent PCNL performed by one single surgeon (Dr. G.J.M.) were included in this retrospective study. All patients had computed tomography (CT) scans and IVP before surgery. Normal preoperative coagulation and negative urine cultures were verified.

All percutaneous accesses were performed under general anesthesia and in a prone position after retrograde ureteral catheterization. Access to the selected calyx was performed by Dr. G.J.M with the aid of ultrasound guidance by using an 18-gauge needle. The tract was dilated with serial dilators from 8F to 20F sheath. An 18F nephroscope (Wolf) was used to inspect the sheath, and we used a holmium laser to fragment stones with the power ranging from 60 to 90 W. Every case was demanded to place an internal ureteral stent on a suspect for the presence of mobile residual stones. A 14F nephrostomy tube was placed in the renal pelvis or the involved calyx for most patients.

Antibiotic prophylaxis was used with the second-generation cephalosporin. The medication was completed after the nephrostomy tube was removed.

Plain radiography of the kidneys, ureters, and bladders was obtained from postoperative day 1 to day 3, according to the state of the patient. The nephrostomy tube was removed when there were neither stone residues nor clinically insignificant residual fragments (diameter less than 4 mm). (Harraz et al., 2017).

All patients were asked to take out the stent for outpatient service 1 or 2 months after the surgery. If there were residual stones, they would have repeated PCNL, ureteroscopy, and shock wave lithotripsy (SWL). After that, all patients were evaluated with an ultrasound test or non-contrast CT scan after 3–6 months postoperatively. All patients accepted follow-ups for at least 1 year. PCNL was considered successful when the patient was stone-free or did not need any further intervention [clinically insignificant residual stone fragments (CIRF)] (Rassweiler et al., 2000).

### Machine Learning Methods

Four types of supervised machine learning algorithms (Lasso logistic, random forests, SVM, and Naive Bayes) were applied in this study. A set of input variables comprising individual variables (age, sex, hypertension, diabetes, hyperlipidemia, urinary infection, renal insufficiency, preoperative hemoglobin, use of anticoagulants or antiplatelet medications, renal and stone factors (previous surgery, stone burden, stone location, and hydronephrosis), surgical factors (postoperative fever, septicemia, need for transfusion, length of stay, stone-free status, and ancillary procedures)) were included. The results of the stone-free status were entered as binary values: 1 (stone residues) and 0 (clinically insignificant residual stone fragments).

**FIGURE 1:**
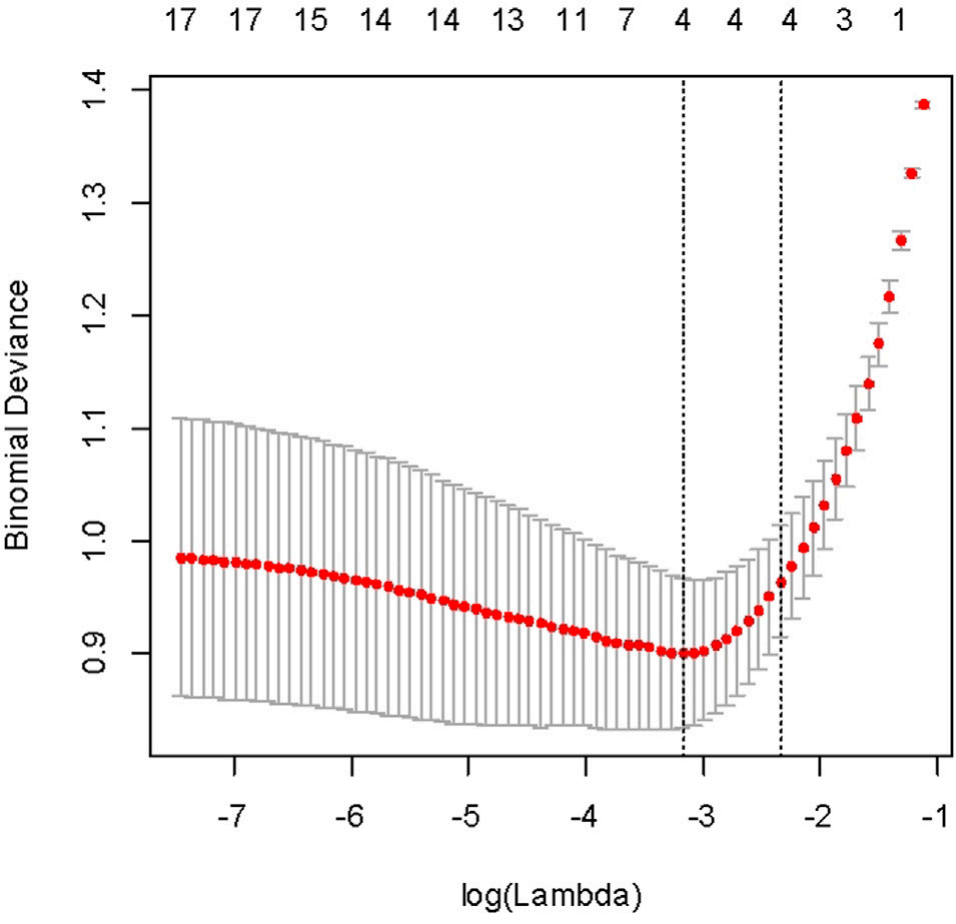
Selecting lambda to screen characteristic variables.

The machine learning models were fitted using scikit-learn 0.18 modules of Python throughout this study. Using lasso regularization and cross-validation (*n* fold = 10) to select the best regression, we selected lambda with 1se.lambda to screen characteristic variables. The selected variables include stone size, stone location (top/middle/bottom), and a total of four variables (Figure 1).

The original data set is randomly divided into the training set and the test set at 7:3 (156: 66). Lasso-logistic, SVM, and Naive Bayes considered the results of lasso regression screening as independent variables to establish a model and calculate the prediction accuracy.

The RF model is a machine learning model built on decision trees. In the decision tree, each node of the tree splits the data into two groups using a cutoff value within one of the features. The RF method can minimize the effect of the overfitting problem by creating an ensemble of randomized decision trees, each of which overfits the data and averages the results to find a better classification.

### Statistical Analysis

Continuous variables were compared using the independent sample Student’s t-test. The model performance was evaluated using the area under the receiver operating characteristic (ROC) curve (AUC), which provides a measure of the discriminatory performance of the model. Sensitivity is the proportion of true positives that are classified as such; specificity measures the proportion of correctly identified true negatives; and accuracy is the proportion of correct predictions.

## Results

**FIGURE 2:**
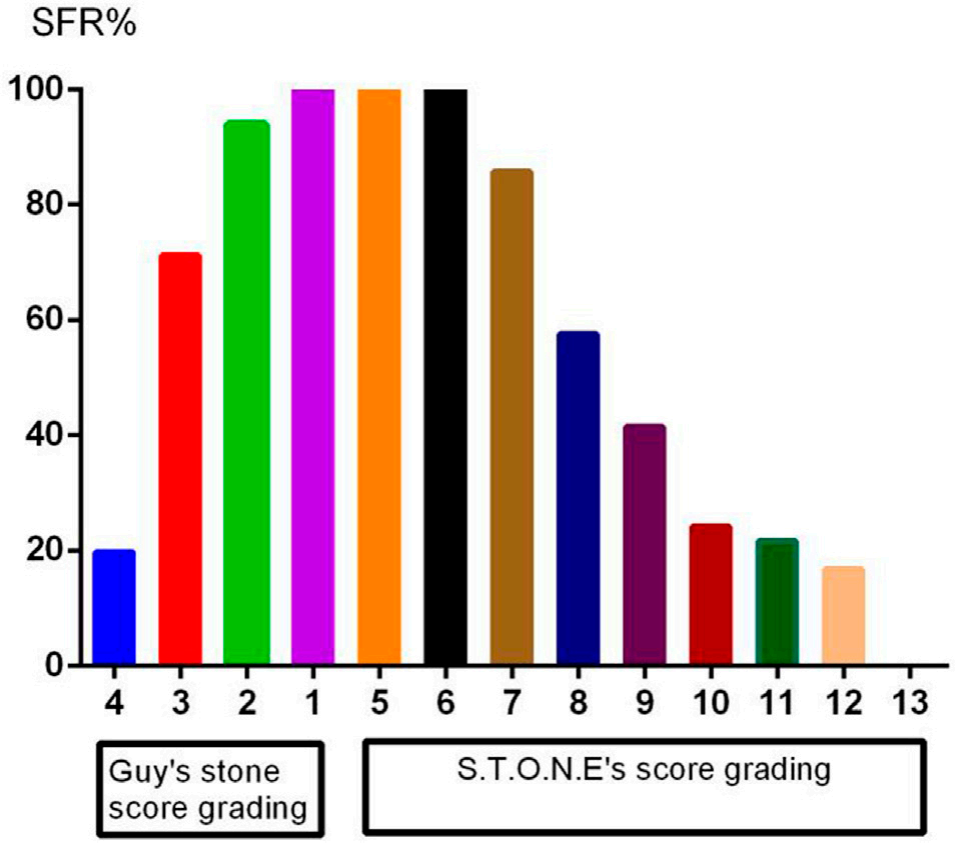
The stone-free rate in each subgroup of GSS grades and the S.T.ON.E score systems.

A total of 222 patients (132 males, 59.5%) were enrolled. The mean age was 54.8 ± 13.3 years, and the mean stone burden was 563.4 ± 517.6 mm^2^. The mean Guy’s score was 3.2 ± 0.9, and the mean S.T.O.N.E. score was 8.9 ± 1.8. Table 1 shows the preoperative factors including individual variables and renal and stone factors. Table 2 shows the actual postoperative data for these patients. The overall SFS was 50% (111/222). Figure 2 shows the stone-free rate in each subgroup of GSS grades and the S.T.O.N.E score systems. The number of fever and infections during hospitalization was 18.9% (42) and 8.6% (19). Postoperative blood transfusion due to significant blood loss happened in nine patients (4.1%). With the follow-ups for at least 1 year, there were 12 patients (5.4%) who accepted ancillary procedures to manage residual renal stones.

**TABLE 1:** Preoperative factors include individual variables and renal and stone factors.

Age (mean ± SD) (years)	54.81 ± 13.31	%
Gender (male/female)	132/90	59.46
Guy’s score	3.27 ± 0.87	
S.T.O.N.E score	8.91 ± 1.82	
Stone burden (mm2)^a^	563.4 ± 517.6	
History of diabetes n (%)	45	20.27
History of hypertension n (%)	70	31.53
History of hyperlipidemia n (%)	39	17.57
Solitary kidney n (%)	18	8.11
Renal insufficiency n (%)	30	13.51
Anemia n (%)	29	31.53
Preoperative urinary infection n (%)	111	50.00
Previous surgery in target kidney n (%)	77	34.68
SMWL	22	9.91
URSL	21	9.46
PCNL	24	10.81
Open surgery	26	11.71
Hydronephrosis n (%)	112	50.45
Stone location n (%)		
Upper calyx	116	52.25
Mid calyx	136	61.26
Lower calyx	164	73.87
Renal pelvis	160	72.07
Ureter	50	22.52

a Stone burden = Length × Width × 0.78.

**TABLE 2:** Postoperative outcome variable (*n* = 222).

Hospitalization day	11.15 ± 4.98	10.49 (%)
Transfusion n (%)	9	4.1
Fever n (%)	42	18.9
Septicemia n (%)	19	8.6
Interventional therapy n (%)	3	1.4
Pleural injury n (%)	2	0.9
Ancillary procedures n (%)	12	5.4
Stone-free rate n (%)	111	50.0

**FIGURE 3:**
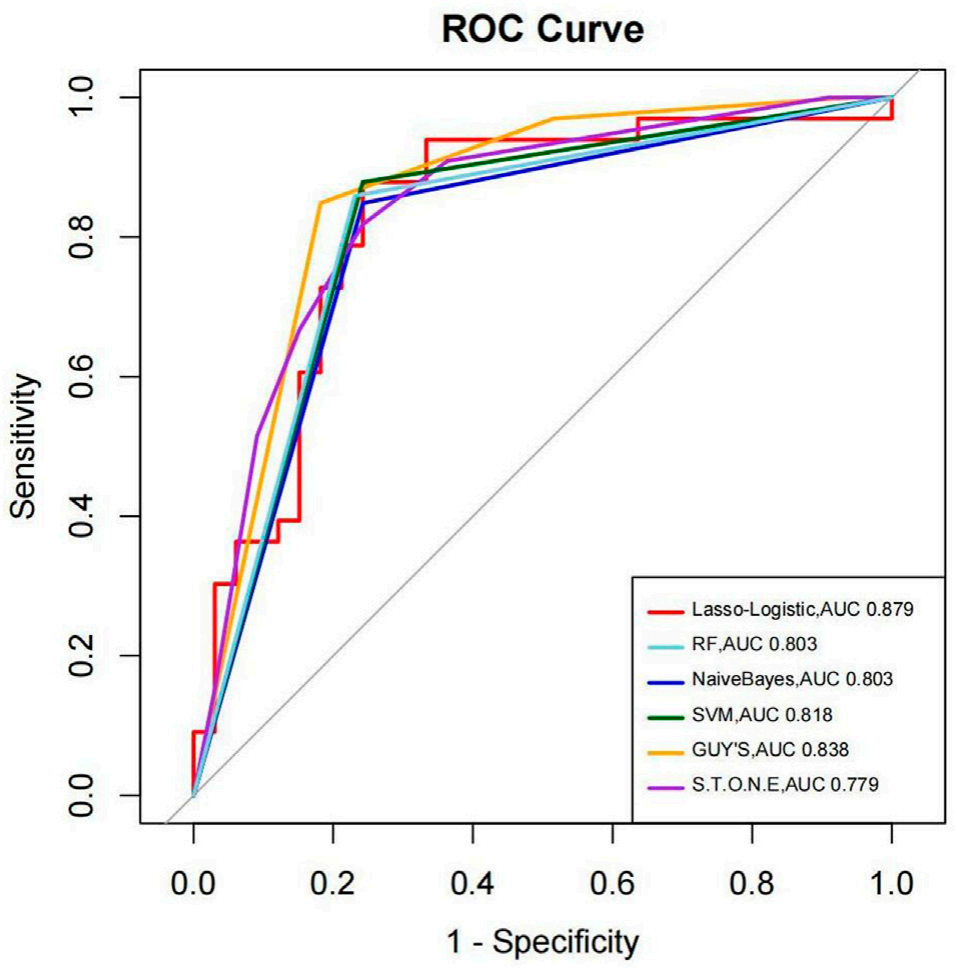
The ROC curves of the four MLMs as well as the GSS and S.T.O.N.E score system.

We have used four machine learning methods to analyze the outcomes to predict the stone-free status. Table 3 shows the AUC, sensitivity, specificity, and accuracy of each prediction method to the results of the stone-free status. When using AUC as a measure of the predictive model performance, as shown in Table 3, the AUC of Lasso logistic was 0.879. It was superior to those of RF, SVM, and Naive Bayes (0.803, 0.818, and 0.803, respectively). The AUCs of the GSS and S.T.O.N.E were 0.800 and 0.844, respectively, which were lower than the Lasso logistic. Figure 3 shows the ROC curves of the four MLMs, as well as the GSS and S.T.O.N.E score system.

**TABLE 3:** AUC, sensitivity, specificity, and accuracy of each prediction method for the results of the stone-free status.

Outcome	Lasso logistic	Random forest	Support vector machine	Naive Bayes	Guy’s score	S.T.O.N.E score system
AUC	0.879	0.803	0.818	0.803	0.800	0.844
Sensitivity (%)	0.7576	0.7576	0.7576	0.8333	0.8180	0.7575
Specificity (%)	0.8788	0.8485	0.8788	0.7778	0.8480	0.8181
Accuracy (%)	0.8181	0.8030	0.8182	0.8030	0.8333	0.7878

As shown in Table 3, the accuracies of the four MLMs were also superior to those of the S.T.O.N.E score system. The sensitivities of the MLMs were 75.8–83.3%, which were higher than the S.T.O.N.E. score system. The machine learning system of LL recognized stone burden and stone location as the most highly weighted preoperative factors affecting the post-PCNL-SFR.

## Discussion

The incidence and prevalence of kidney stones have increased by three times over the past 4 decades (Thongprayoon et al., 2020). The prevalence of kidney stones is estimated at about 5–10% in Europe, 4% in South America, and 1–19% in Asia currently (Sorokin et al., 2017; Liu et al., 2018). Without a doubt, kidney stones represent a considerable burden for public healthcare systems.

Thomas et al. (2011) were the first to introduce Guy’s stone score (GSS) to predict the success of the post-PCNL stone-free status (SFS). The model is reproducible, provides quick and easy office-based categorization of renal stones in four grades based on stone shape and configuration, and correlates well with the SFS; however, it fails to take into account the size and density of the stone. The S.T.O.N.E. nephrolithometry scoring system of Okhunov et al. (Zhamshid. et al., 2013) is based on non-contrast CT (NCCT) having five variables; a score of 5–6 (low complexity) has an overall SFS of 94–100%, and a score 9–13 (high complexity) has an overall SFS of 27–64%. Also, greater S.T.O.N.E. scores are associated with a greater estimated blood loss (EBL), longer operative times (LOTs), and increased length of stay (LOS) in hospital. Smith et al. (2013) developed the CROES (Clinical Research Office of the Endourological Society) nomogram to predict the SFS after PCNL based on a global database study of 5,830 patients. Six characteristics (stone burden, number, location, multiple, staghorn, and institute-level case volume) are included in this nomogram. It achieved a remarkable prediction accuracy of 76%, but it is laborsome and time-consuming.

Many studies have compared the predictive performance of these score systems in post-PCNL SFR. Most studies have examined the performance of these scoring systems to predict SFR equally but not equally to predict complications. The AUC ranges from 0.63 to 0.853 (Wu and Okeke, 2017), and the different scoring system has its drawbacks or limitations. For example, in Guy’s score system, partial staghorn stone was not clearly defined. The S.T.O.N.E. nephrolithometry scoring system relies solely on preoperative CT. The CROES nomogram requires information that might not be readily available (case volume and treatment history). So one simpler and easier application stone score system is needed nowadays. Alireza and his colleagues (Aminsharifi et al., 2017) were the first to use machine learning methods to evaluate the stone-free rate and complications after PCNL. They used ANN to predict the stone-free rate. The accuracy was 81.0–98.2%. The AUC was 0.861. In 2019, his team (Aminsharifi et al., 2020) reported they used software to predict the SFR after PCNL with the AUC of 0.915. In our study, we used four machine learning methods to predict the SFR of PCNL compared with Guy’s system and the S.T.O.N.E. nephrolithometry system. The machine learning methods (MLMs) include Lasso logistic, random forests, SVM, and Naive Bayes. The AUC of the MLMs was superior than that of Guy’s stone score system. The sensitivity and accuracy of MLMs were superior to that of the S.T.O.N.E. nephrolithometry system.

Machine learning is built on the statistical framework. Different approaches are designed to make the most accurate prediction possible. It has been proved to have a good performance to predict the SFR post-PCNL. Although we did not have an advantageous performance of AUC of 0.915 (Aminsharifi et al., 2020), in this study, we found the MLMs could predict the stone-free rate with the AUC not inferior to that of Guy’s stone score or the S.T.O.N.E score system. The machine learning algorithm mainly includes random forests, decision trees, artificial neural networks, Bayesian learning, and Deep learning. Each approach has its advantage and disadvantage. We have tried four methods to predict the stone-free rate in this study, and all of them got a fairly superior performance, as well as the clinical scoring systems being currently available. Machine learning methods are a good tool to predict the stone-free rate with AUCs after PCNL.

So far, in the field of urinary stones, there have been few studies using machine learning methods to predict operative outcomes or help make operative decisions. As one author commented (Peng et al., 2021), to improve the application of MLMs in uritholiasis, two categories should be considered: first, more people including urologists, statisticians, and computer experts need to be involved in this project; second, more data from different regions or population should be collected for future event prediction. We need to establish, manage, and share a cross-country or nationwide database, through which machine learning or AI would contribute to the field of calculi or other issues in the near future.

## Data Availability

The raw data supporting the conclusion of this article will be made available by the authors, without undue reservation.

## Abbreviations

MLMs, machine learning methods; PCNL, percutaneous nephrolithotomy; LL, Lasso logistic; RF, random forest; SVM, support vector machine; AUC, area under the curve; GSS, Guy’s score system; CROES, Clinical Research Office of the Endourological Society; SFR, stone-free rate; NCCT, non-contrast CT; EBL, estimated blood loss; LOT, longer operative time; LOS, length of stay
